# Unique Specific Jumping Test for Measuring Explosive Power in Young Basketball Players: Differences by Gender, Age, and Playing Positions

**DOI:** 10.3390/sports12050118

**Published:** 2024-04-27

**Authors:** Asaf Shalom, Roni Gottlieb, Pedro E. Alcaraz, Julio Calleja-Gonzalez

**Affiliations:** 1 Department of Sports Science, Universidad Católica San Antonio de Murcia, 30107 Murcia, Spain; asaf.fitness@gmail.com (A.S.); ronigot23@gmail.com (R.G.); palcaraz@ucam.edu (P.E.A.); 2 Department of Physical Education, The Research Center for Sports and Physical Activity, Tel Hai College, Qiryat Shemona 1220800, Israel; 3 Wingate Institute, The Academic College Levinsky-Wingate, Wingate Campus, Netanya 4290200, Israel; 4 Research Center for High Performance Sport, Faculty of Sport Sciences, Catholic University of Murcia, 30107 Murcia, Spain; 5 Department of Physical Education and Sports, Faculty of Education and Sport, University of the Basque Country, UPV/EHU, 01007 Vitoria-Gasteiz, Spain

**Keywords:** performance analysis of sport, physical exercise, physical activity explosive power, unique, age, gender, playing positions, young basketball players

## Abstract

When playing basketball, players are required to have high explosive power, which requires the ability to move in efficient, specific, and game-specific movement patterns that combine both horizontal and vertical abilities. Differences have been seen between young male and female basketball players in this measure. The aim of this study was to examine differences in players’ unique movements by gender, age, and playing positions using a novel test for basketball players. This study included 232 young basketball players, male and female, from a range of Israeli leagues, who were divided into three categories: under-14, under-16, and under-18. Our findings showed that males presented better results than females in all age categories. Moreover, females in the under-18 category presented better results than those in the under-14 category, but not more than those in the under-16 category. Differences in playing positions were only examined between males and females in the under-18 category, where players begin to specialize in playing positions, and here, guards showed better results than forwards and centers. Our conclusions highlight the importance of including unique, sport-specific tests in talent identification and selection processes, as these tests can provide valuable information about a player’s skill set and potential for success. The findings are presented in an achievement table of the expected physical fitness results by age and gender for the benefit of basketball coaches and strength and conditioning coaches when assessing their players.

## 1. Introduction

Although basketball is not a new sport, its rules have changed over time. In the modern game, a high level of explosive leg power is essential for players to achieve top-level performance on the court [1,2]. This requires the use of the anaerobic alactic energy system, which enables intense actions over short periods [3,4,5]. Basketball involves many anaerobic actions, such as sprints, jumps, and changes of direction [1]. The ability to perform these actions depends on the player’s anaerobic alactic energy resources, which come from the adenosine tri-phosphate–creatine phosphate (ATP–CP) system stored in the muscles [5,6]. Glycolysis also contributes to anaerobic activities that last longer than a few seconds [1,5,6]. Aerobic energy is also important for fast recovery and the repetition of high-intensity anaerobic actions [7,8,9].

Basketball involves many important movements that require lower limb explosive power, including vertical movements like rebounds and jump shots, horizontal movements like sprints and changes of direction, and combinations of the two like layups [1,3]. These movements are performed intermittently throughout the game [1,2,6].

Explosive power is highly valued by coaches in basketball, and they focus on improving this skill in players of all ages, experience, and levels of performance [10]. To effectively develop players’ explosive power and tailor training programs and game plans, coaches require consistent and accurate tools for assessing players’ explosive power development [2,5]. These tools must be tailored to the specific needs of basketball [1,6].

Basketball demands a unique blend of both horizontal and vertical explosiveness from its players. To evaluate these crucial aspects, various tests have been developed specifically for basketball players [6]. These tests provide coaches and physical therapists with essential data for player analysis and performance enhancement. The following presents an overview of seven tests used for basketball players:

The 5/10 m Sprint Speed Test: This test assesses horizontal explosive power through cyclical movement, measuring how quickly a player can sprint from a standing start over distances of 5 and 10 m. Photo-electric cells provide precise timing. Coaches use the best time from two sprints to gauge a player’s sprinting ability and acceleration [1,2,6].

The Standing Broad Jump Test: An evaluation of anaerobic alactic capabilities, the standing broad jump measures how far a player can jump from a standing position. Players generate momentum by bending their knees and moving their arms before jumping. The best jump out of three attempts is recorded. This test is used mainly in clubs that lack advanced equipment [6,11,12].

The Drop Jump Test: Conducted as either a horizontal or vertical drop jump, this test measures an athlete’s stretch-shortening cycle ability. Athletes stand on a box and drop down before immediately rebounding with either a horizontal or vertical jump. The objective is to minimize ground contact time, emphasizing rapid force production. This test aids in assessing and enhancing an athlete’s ability to utilize elastic energy during quick movements [6,13,14,15].

The 2 × 5 m Change-of-Direction Ability Test: Designed to measure anaerobic alactic capabilities and agility specific to basketball, this test evaluates sprinting, turning, and changing direction. Players sprint 5 m, perform a quick turn, and return to the starting point, simulating the dynamic movements required in a game. This provides insights into a player’s ability to react quickly to changing game situations and make rapid decisions [1,2,6].

The Countermovement Jump Test: Assessing vertical jump explosive power, this test involves athletes bending their knees before jumping as high as possible. By minimizing upper limb momentum, coaches can accurately measure lower body force and power generation. It is a crucial tool for evaluating a player’s ability to elevate themselves quickly during game situations [1,2,6,16].

The Squat Jump Test: Similar to the countermovement jump, this test measures vertical explosive power. Players jump from a low squat position without any pre-movement, focusing solely on lower body force production. Coaches use this test to assess players’ ability to generate power without the aid of a countermovement [6,17].

The Bounding Power Test: Combining horizontal and vertical assessments, this test evaluates anaerobic alactic abilities. Players jump horizontally on one leg as far as possible, alternating legs for six consecutive jumps. The longest distance achieved is recorded, providing insight into players’ overall power and coordination [2,16,18].

### 1.1. Differences in Explosive Power by Gender and Age

Prior research has established that, in the context of basketball, males typically exhibit an elevated quantity of type II muscle fibers, increased muscle mass, superior strength, and enhanced muscle quality as compared to their female counterparts [19,20,21]. These unique traits impact their capacity to execute explosive movements demanding elevated contractile force and speed [6,21,22]. Age also contributes to these variations, as athletes develop and mature over time [10,22]. A study by Ziv and Lidor (2009) on young basketball players illuminated the influence of age and gender in determining lower body strength [21]. In the 11–13-year-old age group, the study reported no significant differences. However, in the 15–17-year age range, distinctions emerged in lower body force, revealing lower values in relative strength among female players relative to their body mass [10,21].

An analysis of the literature highlights a connection between the production of horizontal and vertical forces, as well as their amalgamation, influencing the development of explosive power in basketball players [1,6]. Nevertheless, it is crucial to emphasize that the proficiencies associated with explosive strength and the integration of distinct movement requirements in these dimensions not only apply to the various age categories of basketball players (U14, U16, and U18) but also to distinctions between male and female players within these age cohorts [10,20,22].

### 1.2. Differences in Explosive Power by Playing Positions

Basketball necessitates the execution of distinct skills, movements, and physiological demands, which vary according to the player’s position [6,23]. Existing research indicates that different positions in basketball are associated with distinct physiological requisites, which may also be influenced by age and gender [6,21,23,24]. Anaerobic power and explosive power, particularly in vertical jump performance, have been explored frequently in previous studies [6,21]. Therefore, when formulating training programs, coaches should consider the distinctive physical attributes of players based on their playing positions [24]. Ziv and Lidor (2009) found that forwards typically exhibit smaller and lighter body frames in comparison to centers, yet they possess larger and heavier body frames when contrasted with guards [21]. Furthermore, guards generally demonstrate elevated levels of aerobic fitness compared to players in other positions, as evidenced by field tests such as the YoYoIR1 [21,23]. In contrast to centers, guards manifest superior vertical jumping abilities, whereas centers are characterized by heightened levels of muscular strength and power [21]. Only a handful of studies have evaluated these attributes in a substantial player cohort or during the regular season [9,23,24].

Recent research studies indicate notable disparities in explosive power among different positions in professional basketball—guards, forwards, and centers. Guards exhibit significantly greater explosive power compared to forwards and centers [6,24]. Ziv and Lidor’s (2009) study produced mixed results regarding vertical jump and jumping power differences among basketball players in various positions [21]. While no significant variances were reported in these attributes across positions, guards and forwards displayed notably higher vertical jump heights than centers. The demands of playing positions in basketball differ in terms of anaerobic and explosive power, particularly in vertical jump performance [21,23]. Additionally, age and gender contribute to these physiological demands, with older and male players tending to exhibit higher anaerobic and explosive power [6,20]. Nonetheless, further research is imperative to comprehensively understand the physiological differences among young basketball players based on positions, ages, and genders.

The objective of this study was to assess the explosive power of young elite basketball players using a jump test that is specific to basketball players. This jump test includes the use of a ball and a combination of horizontal and vertical movements. This study aimed to analyze potential differences in explosive power based on age, gender, and playing position, while utilizing a reliable and validated test specifically designed for basketball players [16].

## 2. Methodology

### 2.1. Participants

This research included 232 young basketball players, both male and female, from four clubs in Israel. The study participants were determined by the availability of fit players from four elite basketball teams and individual elite players from the same age group playing in the highest league for their age group. All the participants belonged to the first division league of their age in the country. The study began by taking various physical measurements of each participant, such as height (in meters), body mass (in kilograms), and body fat percentage [25,26]. The height measurement was taken using a stadiometer (SECA, model 213, Hamburg, Germany), whereas body mass and fat percentage were measured using electronic scales (Tanita BC 418, Tanita Corp., Tokyo,, Japan) [27,28]. Measurements were carried out by two of the researchers of this study (A.S and R.G) with the professional staff of each age group, according to the accepted standards for measurements in the country. The anthropometric data appear in Table 1. All the participants had been playing basketball for three to eight years. Additionally, they were required to attend at least two fitness practices, participate in three to five basketball practices, and one league game each week. Finally, inclusion in the study required that players were not suffering from current injuries or aches and were not taking medication. This study was conducted in accordance with the Declaration of Helsinki and approved by the Ethics Committee at the authors’ affiliated academic institution (Reference number: 407, 28 March 2022).

### 2.2. Procedure

After the basketball clubs and coaches were contacted to participate in this study, the players and their parents were requested to provide informed consent. It was made clear that participation was optional for all participants. Although complete anonymity could not be guaranteed due to the study’s nature, all participants and parents were guaranteed the highest level of confidentiality and scientific precision throughout the study. It was emphasized that the data collected would only be used for the research project. This study was conducted during the competition season when the players were at their peak physical fitness, and dates for conducting the study at each club were scheduled to avoid disrupting their training and competitions.

To avoid any variations caused by the time of day, all participants completed the test at 6 pm under standard ambient conditions, at a temperature of 23.1 ± 0.5 °C and a relative humidity of 70.5% ± 3.5%. The assessments were conducted by the researchers and the team’s coach on official indoor basketball courts, and the players were instructed to wear their regular sportswear and basketball shoes. Before the assessments, the players were advised to avoid consuming caffeine, other stimulants, alcohol, and other depressants, and to refrain from strenuous physical activities for at least 24 h. The participants received comprehensive instructions from the study team before participating in the study.

### 2.3. Tools

#### The New Unique Test for Basketball Players

**Figure 1:**
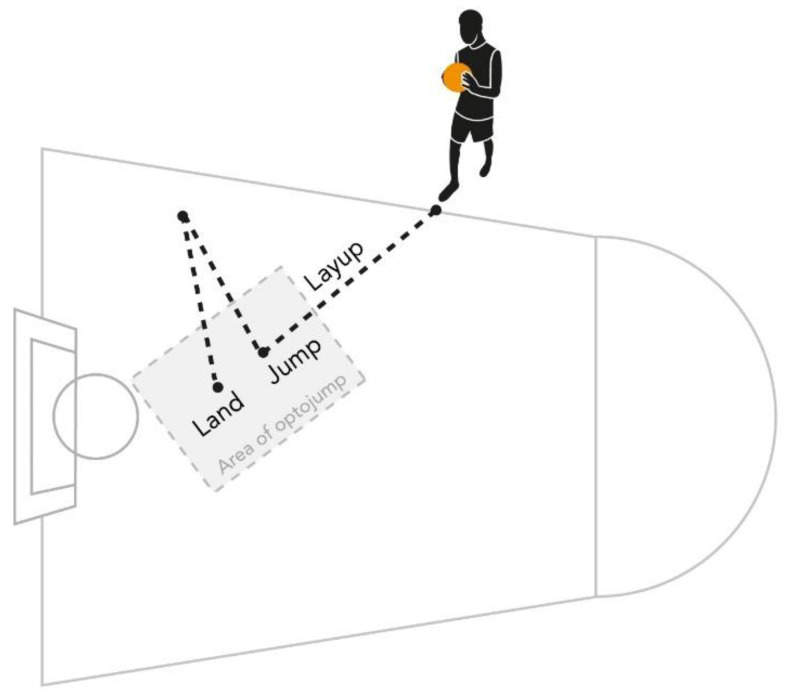
Performance of the new unique test for young basketball players.

Using their preferred hopping leg, players were required to perform penetration and a layup as illustrated in Figure 1. The test entailed a combination of activities such as running, jumping, landing, and releasing the ball toward the basket [16]. It should be noted that the test was conducted on a standard basketball court.

**Figure 2:**
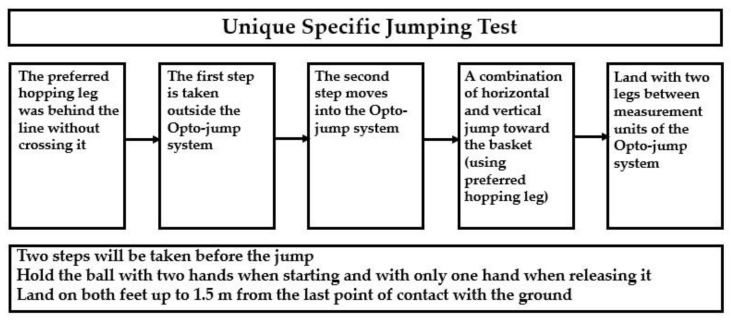
Flow chart of the novel jumping test for young basketball players.

To begin the test, participants stood outside of the Opto-jump system’s detection area, which was located on the painted floor (Optojump System by MicroGate). They held the ball with both hands, then performed a layup into the testing area before executing a combined horizontal–vertical jump while releasing the ball toward the basket using only one hand. At the peak of their jump, they released the ball and aimed it towards the basket. Finally, they landed within the measuring area, no more than 1.5 m from their previous point of contact prior to takeoff. Figure 2 provides a detailed explanation of the flow of the test [16].

In this study, the test was conducted by two basketball coaches and two fitness coaches who ensured that the participants followed the following guidelines: (1) starting behind the foul line without crossing it; (2) taking two steps before the jump; (3) performing a push-off with one leg (the preferred hopping leg); (4) holding the ball with both hands when starting and with only one hand when releasing it; (5) releasing the ball in such a way that it enters the basket or touches the rim after it leaves the player’s hand; (6) landing on the balls of their feet without excessive bending of the knees and landing only on their feet; (7) landing with both feet within the measuring zone; (8) not touching the basket rim or net with the hand during the jump, either before or after releasing the ball; and (9) ensuring the ball did not fall onto the Opto-jump measurement units before the player landed. Players who did not follow these guidelines were asked to repeat the jump. To perform the layup for the test, players were asked to jump as high as possible, performing a horizontal run followed by a vertical jump with horizontal elements. They were instructed to land on both feet within 1.5 m from their last point of contact with the ground after holding the ball in one hand to replicate realistic penetration to the basket. Actually succeeding in shooting a basket was not part of the test.

### 2.4. Variables

The following three independent variables were addressed in this study, including (1) gender (male/female); (2) three age groups (according to their affiliated basketball team): under-14 (U14), under-16 (U16), and under-18 (U18); and (3) three positions groups: guards, forwards, and centers (all from the group of under-18). It should be noted that the U14 group included players aged 13–14, the U16 group included players aged 15–16, and the U18 group included players aged 17–18. Thus, the youngest players were aged 13 in the U14 group.

## 3. Statistical Analysis

In this quantitative study, the prerequisite for conducting statistical tests included thorough verification of the parametric assumptions. Prior to utilizing the statistical techniques, we confirmed the normality of the distribution, homogeneity of variances, and the independence of observations through appropriate tests. The results supported the use of parametric methods, enabling robust statistical analysis. Means and standard deviations (SDs) were calculated for weight, height, and body fat; independent *t*-tests were conducted for age and gender, and 2-way ANOVA tests were conducted to compare mean differences between the age groups, gender groups, and position groups. All statistical analyses were performed using the SPSS v.21 software (Inc., Chicago, IL, USA), ensuring the integrity and validity of the results. Statistical significance was set at *p* < 0.05, aligning with conventional criteria for assessing the presence of statistically meaningful differences.

## 4. Results

Table 1 presents the participants’ physical characteristics, including weight, height, and body fat by age group and gender.

Table 2 presents the participants’ descriptive data by gender (including age and the average of jump height achievement in the unique specific jumping test) and the optimal results of this unique test. Significant differences were seen between genders in their mean jump height achievement, regardless of age, where mean jump height achievement for males (40.82 ± 8.03) was significantly greater than for females (32.76 ± 5.54). Moreover, improvement in these results in line with increased age was also evident, where older players jumped higher.

**Figure 3:**
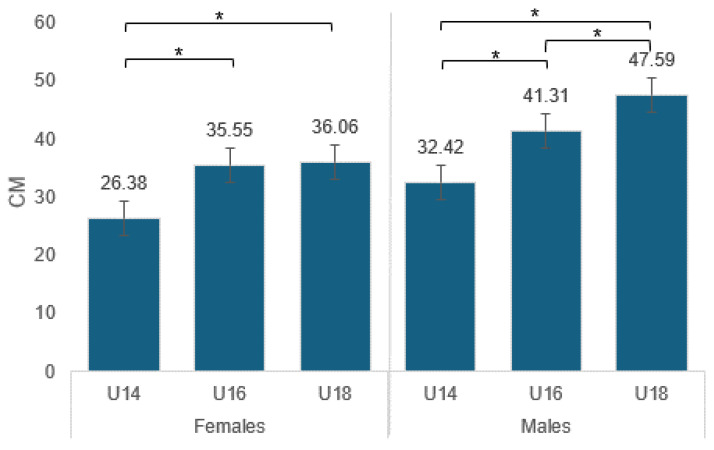
Average jump achievement by age and gender. * Males U18-U14 and U18-U16 and U16-U14 (*p* < 0.05). * Females U14-U18 and U14-U16 (*p* < 0.05).

As seen in Figure 3, there are significant differences between males and females also in relation to age. In the male group, there are significant differences between the age groups U18-U14 and U18-U16 and U16-U14 (*p* < 0.05). In the female group, there are also significant differences between the age groups U14-U18 and U14-U16 (*p* < 0.05).

U14: The effect size (Cohen’s d = 0.41) suggests a moderate difference between males and females in the unique specific jumping test. This suggests that while there is a noticeable difference between the groups, it might not be large in a practical sense for all contexts.

U16: The effect size (Cohen’s d = 0.19) is small, implying a less pronounced difference in jumping test performance between males and females in this age group.

U18: The effect size is large (Cohen’s d = 1.12), indicating a very significant difference between males and females in their jumping test scores. This suggests a difference that is significant both statistically and practically.

**Figure 4:**
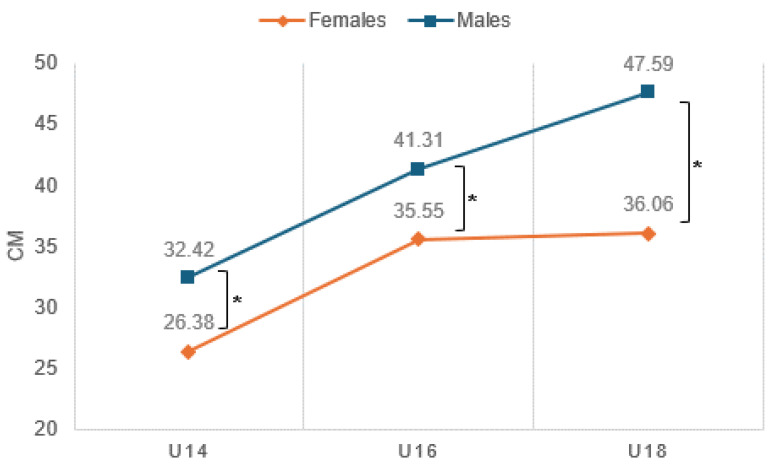
Differences in average jump achievement by age and gender interactions. * Between males–females in U14 and U16 and U18 (*p* < 0.05).

In addition, interactions were also seen between age and gender, where improved jump height in the female participants began to decrease after the age of 16, unlike the continued increase seen in males at the same ages (Figure 4).

Male participants showed consistent significant improvement in mean jump achievement by age, with a significant increase from U14 (32.42 ± 3.83) to U16 (41.31 ± 6.72) and from U16 to U18 (47.59 ± 4.24). Among female participants, on the other hand, no such consistency was seen, with an increase from U14 (26.38 ± 3.42) to U16 (35.55 ± 3.29) but with no significant change from U16 to U18 (36.06 ± 3.30), as depicted in Figure 4. In addition, significant differences were seen in gender in all age groups, between boys–girls in U14 and U16 and U18 (*p* < 0.05), where the jumping performance of males was significantly higher.

**Figure 5:**
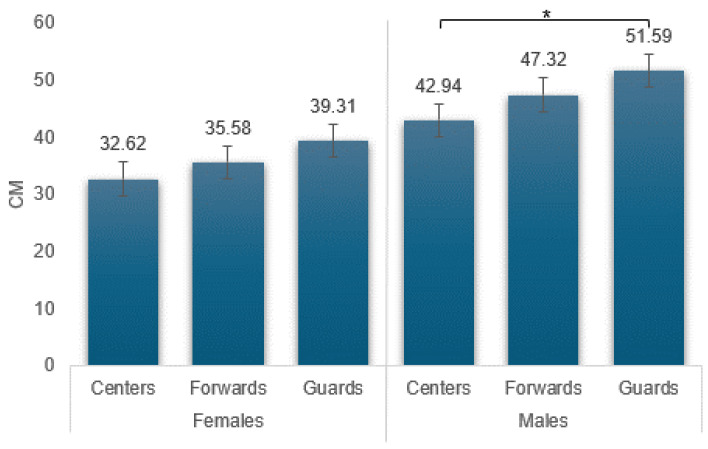
Average jump achievement by gender and playing position in age U18. * Males: guards–centers (*p* < 0.05).

**Figure 6:**
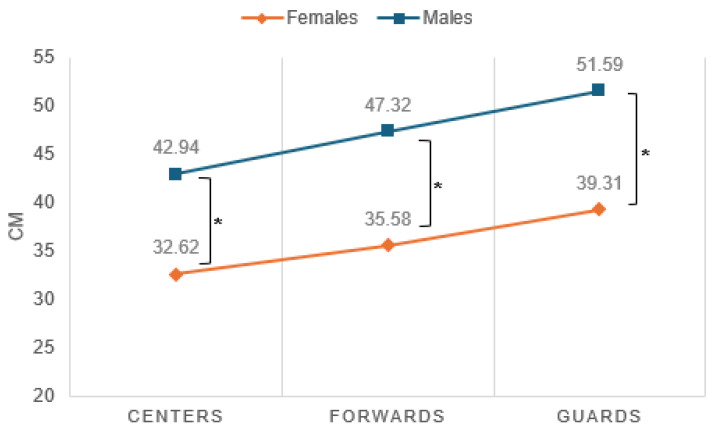
Differences in average jump achievement by gender and playing position interactions. * Between males–females in guards and forwards and centers (*p* < 0.05).

When examining differences in gender and playing positions among the U18 age groups, only the male groups showed significant differences between playing position, as depicted in Figure 5. The guards jumped significantly higher than the centers. In addition, as seen in Figure 6, there were significant differences in gender in all positions of the game.

## 5. Discussion

High-level explosiveness is crucial to the performance of young basketball players [1,6]. This component is dependent on genetics but can also be developed through various training programs [2]. In order to compete at high levels in basketball, players require specific and unique abilities for the game [1,2,6,9,16]. The game demands a combination of horizontal and vertical explosiveness [1,2,6,16]. Young players who are able to express their explosiveness in specific movements of the game will have a significant competitive advantage over players with lower explosiveness levels [2,5,29].

The new test we developed measures a basic basketball movement that is first learned when starting basketball but is constantly practiced and used in games at all ages and levels while holding a ball [6,16]. It is important to note that using the ball during the test is not a limitation and may even be beneficial for specifically assessing the jump among basketball players. The skills required for the test are relatively complex, involving explosive power on both horizontal and vertical planes while holding a ball [16]. However, for elite basketball players, these skills are basic and frequently used in warmups, practice, and games. That is why we only tested highly experienced basketball players from elite clubs to ensure that they had excellent control over the movement being examined.

As was explained above, successful performance by basketball players depends on their anaerobic alactic system, with shorter and more intensive actions requiring greater explosive power [1]. As such, it is important to develop training processes and specific tests for this factor. At the same time, it is also important to understand the more frequent movements required of basketball players in many situations [2,6]. During practice and games, key actions include vertical, horizontal, and combined vertical and horizontal movements. Professionals in the game consider optimal training methods to develop these physical abilities among the players, especially explosive power [1,2,6]. The first objective of this research was to examine differences in specific explosiveness based on gender and age groups, using a unique and innovative test for basketball players presented in this study. The test simulates a specific movement of basket penetration with a ball. Significant differences were found between genders, where male players had higher average vertical jump heights compared to female players in each age group. Significant differences were found in the effects of age on performance between genders and within groups of female players. Male players exhibited consistent improvement with age, whereas female players displayed a different pattern. Although female players showed improvement between ages U14 to U16, similar to male players, no significant improvement was observed between ages U16 to U18 in female player groups. This may well be connected to maturation and to the significant increase in fat percentages among females at this age, in contrast to males, as can be seen in Table 1. This finding is in line with the study that reported different effects of gender on the adolescent age and their implications for sports performance [30].

The second objective of this study was to examine differences in specific explosive power ability using a unique test [16] based on gender and playing positions [6]. Since younger basketball players typically play in all positions and not in specific ones, this study examined differences in playing positions in only male and female U18 groups. Significant differences were found between genders, as male players had higher average vertical jump heights than female players in all playing positions. These findings are also in line with previous studies that investigated the effect of gender on playing positions in basketball [21,23,24]. Additionally, significant differences were found between playing positions in only the male participant group, where guards achieved significantly higher results in the specific unique jumping test compared to centers.

## 6. Limitations

Despite its practical and theoretical contributions to the field, this study has some limitations that should be addressed. This study was initially conducted during the competition season when the players were at peak fitness; therefore, the findings may be less relevant to other periods. Additionally, this study was only conducted on young basketball players from four competitive clubs, and it is important to verify the data presented in this study on adult players, particularly regarding game positions. Future studies should examine the impact of the new and unique test on basketball players at lower than elite leagues, as this test only examined players in the highest league (the first division league of their age group in the country).

## 7. Conclusions

In conclusion, our study sheds light on the differences in performance of a novel specific jumping test among young basketball players, with age, gender, and playing position all affecting the results. Our findings also provide an additional reason for using a specific test in the field to measure movement that integrates both the horizontal and vertical planes in movement with a ball. The research literature indicates a need for developing specific tests [1,6], and the one presented here provides an additional tool for use in the field.

Our findings highlight the importance of including unique, sport-specific tests in talent identification and selection processes, as these tests can provide valuable information about a player’s skill set and potential for success in sport, and in basketball in particular. Furthermore, coaches and trainers should consider these factors when designing training programs to improve jumping ability, as individualized approaches may be necessary for optimal development. Future research should continue to explore the effects of other factors, such as training history and physical fitness, on performance in regard to specific jumping tests for young basketball players. Ultimately, a better understanding of the unique characteristics of young basketball players can help optimize their athletic development and enhance their potential for success.

## 8. Practical Applications

The findings from our study have led to the creation of an Estimated Achievement Table (Table 3) that can be utilized by coaches and trainers of young basketball players. This customized scale accounts for the age and gender of the players and provides an estimated jump performance score using the Unique Specific Jumping Test for Basketball Players. The results were divided into five groups of percentiles. Each group was divided according to their score level. With this tool, coaches and trainers can rank their players’ jump performance on a scale from unprepared to excellent. However, it should be kept in mind that the data collected in this study pertain to the peak of the sports season, and thus, the data in the table may not be applicable to other stages of the competitive cycle.

## Figures and Tables

**Table 1:** Participants’ physical characteristics by mean (±SD).

FAT%	Height (m)	Body Mass (kg)	N		
10.83 ± 1.3	1.86 ± 5.3	76.4 ± 7.5	42	U18	
10.62 ± 1.3	1.78 ± 6.7	65.9 ± 8	37	U16	Males
11.01 ± 1.3	1.73 ± 6.9	58.1 ± 7.9	36	U14	
25.33 ± 4.6	1.66 ± 5.1	59.8 ± 5.8	42	U18	
23.451 ± 3.4	1.63 ± 4.8	56.9 ± 5.7	37	U16	Females
22.95 ± 5.6	1.58 ± 4.8	48.2 ± 4.4	38	U14	

**Table 2:** Participants’ descriptive statistics.

Optimal Results (cm)	USJT (cm)	N	Age	Gender
41.21 ± 2.57	32.42 ± 3.83	36	U14	
51.21 ± 0.73	41.31 ± 6.72 *	37	U16	Males
56.11 ± 1.09	47.59 ± 4.24 *	42	U18	
31.48 ± 0.55	26.38 ± 3.42 *	38	U14	
41.36 ± 1.03	35.55 ± 3.29	37	U16	Females
41.77 ± 0.40	36.06 ± 3.30	42	U18	

* Significant differences between age groups *p* < 0.05. USJT = unique specific jumping test (the new test), USJT (cm) = the averages of each group; optimal results = the averages of the three best results for each group.

**Table 3:** Achievement Table (Unique Specific Jumping Test for Basketball Players).

Achievement Table (USJT)					
BOYS			GIRLS		
Age	High (cm)	Assessment	Age	High (cm)	Assessment
U18	>49.87	Excellent	U18	>38.87	Excellent
	(46.66–49.87)	Very good		(35.70–38.87)	Very good
	(43.10–46.66)	Good		(32.97–35.70)	Good
	(41.54–43.10)	Poor		(31.44–32.97)	Poor
	<41.54	Unprepared		<31.44	Unprepared
Age	High (cm)	Assessment	Age	High (cm)	Assessment
U16	>47.33	Excellent	U16	>36.77	Excellent
	(43.33–47.33)	Very good		(33.34–36.77)	Very good
	(36.34–43.33)	Good		(31.34–33.34)	Good
	(32.89–36.34)	Poor		(26.88–31.34)	Poor
	<32.89	Unprepared		<26.88	Unprepared
Age	High (cm)	Assessment	Age	High (cm)	Assessment
U14	>37.77	Excellent	U14	>32.12	Excellent
	(31.88–37.77)	Very good		(26.88–32.12)	Very good
	(27.81–31.88)	Good		(24.27–26.88)	Good
	(23.22–27.81)	Poor		(18.22–24.27)	Poor
	<23.22	Unprepared		<18.22	Unprepared

## Data Availability

The data presented in this study are available on request from the corresponding author and the first author. The data are not publicly available due to ethical and privacy restrictions.
